# Molecular cross-talk via extracellular vesicles for the characterization of young subjects with type 1 diabetes unravels new potential markers of insulin resistance and double diabetes

**DOI:** 10.1186/s13098-025-02042-7

**Published:** 2025-12-10

**Authors:** Maria Concetta Cufaro, Ilaria Cicalini, Paola Irma Guidone, Paola Lanuti, Francesca D’Ascanio, Maria Alessandra Saltarelli, Lorenza Sacrini, Anna Piro, Domenico De Bellis, Gessica Di Carlo, Luca Natale, Serena Veschi, Damiana Pieragostino, Piero Del Boccio, Claudia Rossi, Stefano Tumini

**Affiliations:** 1 Department of Innovative Technologies in Medicine and Dentistry, G. d’Annunzio University of Chieti-Pescara, Chieti, 66100 Italy; 2 Center for Advanced Studies and Technology (CAST), G. d’Annunzio University of Chieti-Pescara, Chieti, 66100 Italy; 3 UOSVD Paediatrics Della Murgia Fabio Perinei Hospital, Altamura (BA), 70022 Italy; 4 Department of Medicine and Aging Sciences, G. d’Annunzio University of Chieti-Pescara, Chieti, 66100 Italy; 5 Department of Humanities Law and Economics, Leonardo da Vinci University, Torrevecchia Teatina (CH), 66010 Italy; 6 Department of Maternal and Child Health UOSD Regional Center of Paediatric Diabetology, Chieti Hospital, Chieti, 66100 Italy; 7 Department of Pharmacy, G. d’Annunzio University of Chieti-Pescara, Chieti, 66100 Italy; 8 Department of Science, G. d’Annunzio University of Chieti-Pescara, Chieti, 66100 Italy

**Keywords:** Double diabetes, Extracellular vesicles, FACS-Proteomics, Acylcarnitines, Insulin resistance

## Abstract

**Background:**

Insulin resistance (IR) is commonly calculated using a simple mathematical formula, the eGDR (estimated Glucose Disposal Rate), but in the paediatric type I diabetes (T1DM) population this value has provided contrasting information. We aimed to provide a clearer metabolic “fingerprint” in children with “double diabetes”, focusing on the molecular cross-talk mediated by extracellular vesicles (EVs).

**Methods:**

Paediatric patients were classified based on the eGDR value in: insulin-resistant (T1DM+, eGDR < 8 mg/Kg/min, *n* = 29) and non-insulin-resistant (T1DM-, eGDR > 8 mg/Kg/min, *n* = 35). Venous blood collected from them, and 30 healthy controls was used to obtain dried blood spots (DBS) for AAs and ACs analysis by FIA-MS/MS and for EV by a patented flow cytometry method. Then, EVs were subjected to shotgun proteomics analysis by LC-MS/MS.

**Results:**

Our data showed that T1DM + EVs were packaged with proteins involved in fatty acid metabolism suppression through *STAT3* inhibition and related to possible liver damage. ACs on DBS samples corroborated these data, demonstrating a significant increase in oleoylcarnitine (C18:1), linoleoylcarnitine (C18:2), and myristoylcarnitine (C14) in T1DM+. The combination of clinical and metabolic data led to the identification of a statistical model with an out-of-bag error of 0.115%, demonstrating that palmitoleoylcarnitine (C16:1) and C18:1 are the metabolites that best distinguish children with T1DM + from T1DM- ones. C16:1 correlated significantly with eGDR (*p* = 0.0023).

**Conclusions:**

Combined “omics” approach allowed us to identify a new metabolic “photograph” in a complex context involving diabetes complications related to obesity and IR in a paediatric population that is not yet fully characterized, identifying EVs as well-organized and functionalized shuttles.

**Supplementary Information:**

The online version contains supplementary material available at 10.1186/s13098-025-02042-7.

## Background

Type 1 Diabetes Mellitus (T1DM) is an autoimmune disorder characterized by pancreatic β-cells destruction and lifelong dependence on exogenous insulin. It is traditionally associated with absolute insulin deficiency, thereby differing pathogenetically from type 2 diabetes (T2DM), in which insulin resistance (IR) and reduced secretion of insulin contribute synergistically to disease development [1]. IR is defined as a diminished biological response of insulin-sensitive tissues to physiologic concentrations of insulin. It typically precedes the onset of hyperglycaemia and constitutes a key early event in the progression toward overt T2DM [2]. A subset of individuals with T1DM develops concomitant IR, a clinical entity referred to as “double diabetes” [3, 4]. This term was first coined in 1991 based on the observation that patients with T1DM, who had a family history of T2DM, were more likely to be overweight and rarely achieved adequate glycaemic control even with higher insulin doses: the more extensive, or stronger, the family history, the higher the dose the patient received [5]. This clinical phenotype is increasingly recognized for its association with obesity, metabolic syndrome (MetS), higher risk of complications such as metabolic associated fatty liver disease (MAFLD), and as a key factor in accelerating the process of atherosclerosis both in the general population and in patients with T2DM [6, 7]. Its prevalence in the paediatric population is increasing, particularly among obese children and adolescents and, in patients with T1DM, IR may predict the risk of macrovascular complications, microalbuminuria, retinopathy, and neuropathy [8–11]. Despite the well-established role of IR in the development of complications in T1DM, its quantification remains challenging. While the hyperinsulinaemic-euglycemic glucose clamp technique represents the gold standard for assessing insulin sensitivity, its invasive nature and complexity limit its applicability in large scale or paediatrics studies [12]. Surrogate markers such as the homeostasis model assessment (HOMA) are not suitable for individuals with T1DM, due to their dependence on exogenous insulin [13]. To address this gap, the estimated Glucose Disposal Rate (eGDR), a validated mathematical model, incorporating clinical variables such as hypertension, glycosilated hemoglobin (HbA1c), and waist-to-hip ratio, has been proposed and applied also in paediatric cohorts with T1DM [14]. However, its application in paediatric cohorts remains controversial, due to the influence of pubertal status and variability in anthropometric parameters [15]. Recent attention has focused on extracellular vesicles (EVs) as both biomarkers and mediators of pathophysiological processes in metabolic diseases [16, 17]. In general, EVs represent a heterogeneous population of membrane-enclosed particles released by virtually all cell types [18–20]. They carry a diverse array of biological cargo, including RNAs, microRNAs, proteins, lipids, and DNA fragments, and are key mediators of intercellular communication [21–23]. Traditionally, EVs have been categorised into three main subtypes based on their biogenesis: exosomes, microvesicles, and apoptotic bodies. Exosomes, typically originating from the endosomal pathway, are released via exocytosis; microvesicles, also referred to as microparticles or ectosomes, bud directly from the plasma membrane of their parent cells; whereas apoptotic bodies are generated during cellular apoptosis [24, 25]. More recently, additional EV subtypes have been identified. These include oncosomes, particularly large oncosomes (1–10 μm in diameter), which derive from membrane blebbing in cancer cells, and migrasomes, vesicle-like structures that form along retraction fibres of migrating cells and are implicated in several biological processes [26, 27]. Despite these distinctions, EV subtypes often exhibit overlapping size ranges and phenotypic features, rendering classification based solely on biogenesis both challenging and potentially misleading [18, 28]. In light of this, the International Society for Extracellular Vesicles (ISEV) has recommended the use of the umbrella term “extracellular vesicles” (EVs) for all such particles. To facilitate a more standardised approach, EVs are currently broadly categorised by size: small EVs (< 200 nm) and medium/large EVs (>200 nm) [18, 28, 29]. In this context, while there has been a significant amount of data published on metabolomic analysis of the development of IR and T2DM in adults, the studies in T1DM and in the paediatric population in general are more limited [30]. In fact, to date, none of the available studies have explored the hypothesis of a metabolic fingerprint in paediatric population affected by “double diabetes”. For this reason, our study aimed to characterize the metabolic signature of children and adolescents with “double diabetes”, with a specific focus on the molecular crosstalk mediated by EVs in the context of in vivo “liquid biopsy”. Our innovative patented approach, in fact, uses a lipophilic cation dye (LCD) to distinguish intact EVs from untouched biofluids (patent codes: 10,201,800,000,398; EP19164567A·2019-03-22). The combination of FACS-Proteomics usually takes into account the key role of EVs in vehiculating functional information related to pathological states, such as diabetic conditions and IR implications in this paediatric diabetes, so it could be useful to better investigate the aetiopathogenesis in this complex scenario together with high performance of metabolic signature obtained on dried blood spots (DBS) through flow injection analysis-tandem mass spectrometry (FIA-MS/MS).

## Materials and methods

### Patient enrolment

All children and adolescents under the age of 18 with T1DM who came to the Regional Service of Paediatric Diabetology, Chieti University Hospital “Ss.ma Annunziata”, for an outpatient control in the period between February 2018 and June 2018 were enrolled. This study was conducted following the protocol approved by the Ethical Committee of “G. d’Annunzio” University, in accordance with the Declaration of Helsinki (World Medical Association, 1997) on 13th June 2019. The following exclusion criteria were applied: diabetes other than T1DM, clinical signs of hyperandrogenism, ongoing steroid treatment, associated diseases, such as celiac disease, autoimmune hypothyroidism, genetic diseases, oncohaematological diseases, neurological diseases with cognitive deficits associated or not with impaired motor skills). For all children and adolescents, involved in the study, peripheral blood samples were drawn using sodium citrate tubes (Becton Dickinson Biosciences-BD, San Jose, CA, USA) with the simultaneous assessment of glycosylated haemoglobin (HbA1c) by immunoassay using the Siemens DCA 2000+ (Siemens, HealthCare Diagnostics, Melbourne, VIC, Australia).

The quantitative variables collected included age, duration of T1DM, weight, height, body mass index (BMI), waist circumference and waist-to-height ratio (WHtR), blood pressure (BP), glycosylated hemoglobin (HbA1c), % coefficient of variation (%CV), mean HbA1c of the last 12 months, insulin requirement (U/kg/day), insulin sensitive factor (ISF, mg/dl), basal insulin daily dose, ultrarapid insulin daily dose, insulin-carbohydrated ratio at meals (I: CHO at breakfast, lunch, dinner), total cholesterol (TC), high density lipoprotein cholesterol (HDL-c), low density lipoprotein cholesterol (LDL-c), triglicerides (TGs), number of first grade relatives with T2DM.

IR was calculated using the following mathematical model of estimated Glucose Disposal Rate (eGDR):

eGDR (mg/kg/min) = 21.158 - (3.407 x hypertension*) - (0.090 x WC, cm) - (0.551 xHbA1c, %).

[hypertension*: yes = 1, no = 0]

According to eGDR levels study population was divided into two groups, the most common value chosen as a reference clamp measure in the validation study: 8 mg/Kg/min [14, 31]. Detailed information about T1DM study population were listed in Table S1. A group of healthy controls (*n* = 30, HCs) was collected to compare the two T1DM clinical conditions by matching sex and age.

### Metabolites analysis by FIA-MS/MS

Whole blood from each patient of the groups involved in the study was collected on filter paper card as dried blood spot (DBS), particularly suitable for small volume samples. DBS samples for each group underwent to a simple extraction with NeoBase™ 2 Non-derivatized MSMS kit (Revvity, ex PerkinElmer, Turku, Finland) for the screening of 57 metabolites, i.e. 14 amminoacids (AAs), 2 nucleosides, free carnitine (C0), 35 acyl-carnitines (ACs), 4 lysophosphatidylcholines, and succinylacetone (SA). Subsequently, flow injection analysis-tandem mass spectrometry (FIA-MS/MS) using the system consisted of ACQUITY UPLC I-Class coupled to a Xevo TQD IVD tandem quadrupole mass spectrometer (Waters™ Corporation, Manchester, UK), operating in ESI^+^ by MRM acquisition, was exploited for evaluating metabolites concentrations. Data were processed by MassLynx™ (IVD) Software V4.2 with NeoLynx™ Application Manager (Waters™ Corporation, Manchester, UK). Both MS parameters and detailed specifications of extraction protocol were reported in our previous works [32, 33], together with the list of the metabolites and their internal standards (ISs).

### Flow cytometry staining of extracellular vesicles

The EV staining was carried out as already published [23, 24, 34]. Briefly, 5µL of blood were added to the mix of reagents detailed in Table S2. To prevent immune complex formation and reduce nonspecific background due to antibody aggregation, each reagent stock solution was centrifuged prior to use (21,000 × g, 12 min). Following a 45 min staining period (room temperature, protected from light, or at 37 °C when Annexin V was not included in the reagent mix), 500 µL of Binding Buffer (BD Biosciences, San Jose, CA, USA) were added to each tube, and 1 × 10^6^ events/sample were acquired by flow cytometry (FACSVerse, BD Biosciences, San Jose, CA, USA). Sample dilution was optimized, and at the working dilution (1:143) no swarm effects were observed. All requirements for polychromatic flow cytometric analysis of EVs were satisfied [35–38]. Analytical and experimental design variables were implemented in accordance with the MISEV, and MIFlowCyt-EVguidelines for flow cytometry–based EV studies [39, 40].

### Isolation of EVs by fluorescence-activated cell sorting (FACS)

For each sample, 5 µL of peripheral blood were stained with 1 µL of FITC-conjugated phalloidin (Sigma) and 3 µL of an APC-emitting LCD reagent (patent codes: 10,201,800,000,398; EP19164567A·2019-03-22, BD Biosciences). Following 45 min of incubation at room temperature in the dark, samples were diluted with at least 500 µL of PBS. Further dilution was performed as needed to prevent swarming artefacts. EVs, defined as LCD+/phalloidin- ents, as already published [19], were isolated by fluorescence-activated cell sorting using a FACSAria III instrument (BD Biosciences) equipped with a 100 μm nozzle. The trigger threshold was set on the APC channel (corresponding to LCD emission), and data acquisition was carried out using bi-exponential scaling and height (H) signals for all parameters. Instrument performance, fluorescence calibration, and data reproducibility were monitored and maintained using the Cytometer Setup & Tracking Module (BD Biosciences). The purity of sorted EVs was assessed over time, and only samples with a post-sorting purity greater than 90% were used for subsequent analyses.

### EV shotgun proteomics analysis

2.9 × 10^6^ total EVs sorted from peripheral blood (PB) of T1DM+, T1DM- and HC subjects were pooled and analyzed for characterizing EV protein outfit. Shotgun proteomics analyses were performed accordingly with methods previously described by our research group [41]. EVs were disrupted by sonication in a lysis buffer (Urea 6 M/Tris HCl 100 mM, pH = 7.5), digested overnight at 37 °C with trypsin (Promega, Madison, WI, USA), and acquired in triplicate by LC-MS/MS using a Proxeon EASY-nLCII (Thermo Fisher Scientific, Waltham, MA, USA) chromatographic system coupled to a Maxis HD UHR-TOF (Bruker Daltonics GmbH, Bremen, Germany) mass spectrometer as already described [41]. Quantitative data analysis of sorted EVs was performed by MaxQuant version 1.6.3.4 (Max-Planck Institute for Biochemistry, Martinsried, Germany), using raw data file of MS/MS spectra against Andromeda peptide search engine [42] using UniProt database (released 2018_04, taxonomy Homo Sapiens, 20874 entries). All processing parameters were set as detailed in Rossi et al. [32]. Two single proteomics analyses were performed for quantifying both differential expressed proteins and unique proteins in two clinical groups obtaining the protein ratios for these comparisons: T1DM+/HC and T1DM-/HC. Intensity-based absolute quantification (iBAQ) in MaxQuant [43] was used to quantify protein abundance in each EV mixture setting the protein quantification with at least one unique peptide and the quantification of the protein in at least two technical replicates for each group. False discovery rate (FDR) at the protein level was set at 3%, on the contrary at peptide level was set at 1%. The whole EV protein data sets were further uploaded for “Core Analysis” through Ingenuity Pathway Analysis system (IPA, Qiagen, Hilden, Germany) in order to perform a “Comparison Analysis” between two clinical conditions normalized on healthy control EV sample.

### EV characterization by nanoparticle tracking analysis

Size and concentration of sorted EV samples were subsequently measured by nanoparticle tracking analysis (NTA) on a ZetaView instrument (Particle Metrix, Meerbusch, Germany) equipped with a 488 nm, 40 mW blue laser and a long-pass emission filter for blue laser excitation, as already reported [44]. The autofocus was calibrated by the system to ensure clear particle visualization. Post-acquisition parameters were optimized and consistently applied across all measurements. For each sample, a minimum of 11 fields were recorded, with 20-second videos captured using the ZetaView software. For every measurement, a comprehensive report was generated, including all analyzed parameters such as the mean and median EV sizes.

### Statistics

After that FIA-MS/MS data were processed using NeoLynx software (Waters, Manchester, UK), levels of AAs, ACs, C0, SA, nucleosides, and lysophospholipids were used for Random Forest algorithm using Metaboanalyst 6.0 software, as well multivariate Receiver Operating Characteristic (ROC) curve that was performed using the Support-Vector Machine (SVM) algorithm as classification method and the SVM built-in as feature ranking method. Moreover, for each metabolite, after removing outliers, D’Agostino and Pearson test was performed for evaluating the normality distribution. If normality test passed, the ANOVA with Tukey’s multiple comparisons test was used, whereas the Kruskal-Wallis test with Dunn’s multiple comparisons test was performed for non-parametric distribution. The same approach was used for the assessment of EV subtypes counts, diameter and size distributions. GraphPad Prism 9.0.0 (GraphPad software, Inc, La Jolla, CA 92037, USA) was used for all statistics analyses. The values of *p* < 0.05 were considered significant. The 95% of confidence interval was assumed for each test. The correlation analysis was performed using MEDCALC Demoversion.

## Results

### Descriptive statistics and clinical data

The general features of the T1DM children and adolescents were shown in Table S1. eGDR stratification generated two groups: T1DM + and T1DM- individuals, the first classified the more insulin resistant group with an eGDR level < 8 mg/Kg/min and the second the group without IR showing an eGDR level > 8 mg/Kg/min. 23 subjects were reclassified in the first group, with an equal distribution of males (53%, *n* = 12) and females (47%, *n* = 11), as well as for prepubertal (47%) and pubertal (53%) subjects (p-value = ns). There was no statistical significance in type of basal/regimen used between the two groups in lipid profile. Moreover, T1DM + patients had significantly higher age, duration of the disease, number of first grade relatives with history of T2DM, BP values and their Z scores, waist circumference, WHtR, weight, weight sds, BMI, BMI sds, and HbA1c (p-value = 0.01). Moreover, T1DM + patients had significantly lower I: CHO ratio at dinner (p-value = 0.043), which means that they administrate more insulin at this meal, as well as significantly higher basal units per Kg and per day. Interestingly, T1DM + patients had lower ISF, but the difference between the two groups was not statistically significant (p-value = 0.084), as well as for the insulin requirement (p-value = 0.11).

### Liver damage in diabetes type 1 with insulin resistance is mediated by extracellular vesicles

**Fig. 1:**
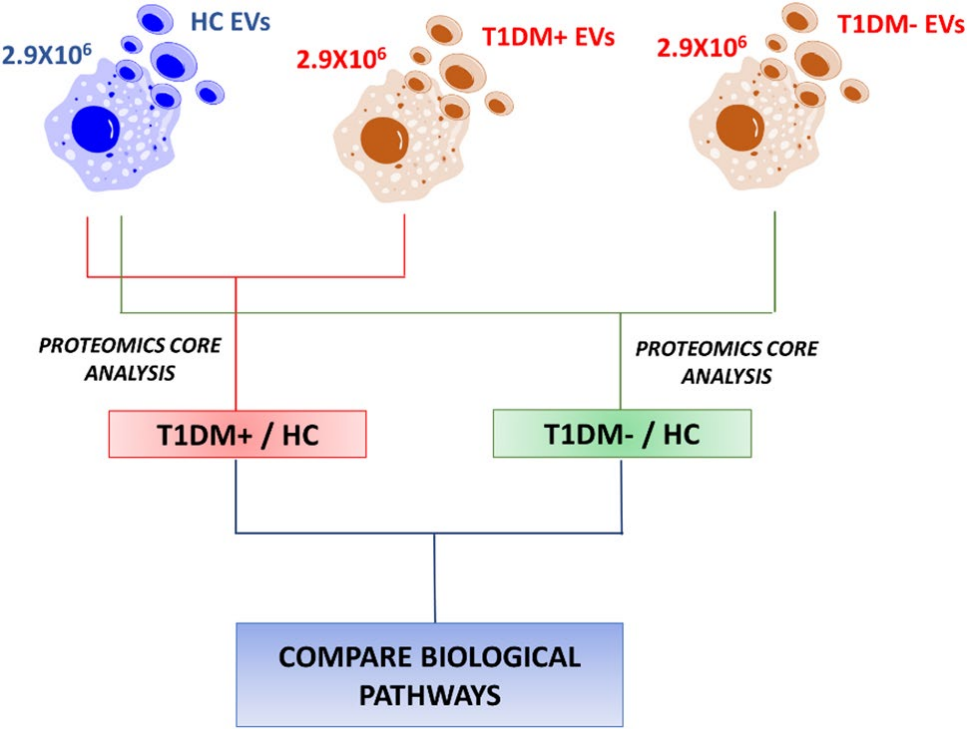
Proteomics workflow scheme. It underlines the proteomics strategy used for EVs characterization by normalizing proteomics results on healthy controls (HC). Two distinct Core analyses were performed by Ingenuity Pathway Analysis (IPA) and then, compared for evaluation of differential modulated pathways

Actually, EVs are considered promising tools for biomarker discovery to pave the way for monitoring and unravelling new pathophysiological mechanisms in many diseases, such as T2DM modulating insulin signalling [45]. A list of markers, as detailed in Table S2, was tested to analyze different EV subtypes. In detail, GLUT-4 is the insulin-regulated glucose transporter was used to identify adipose tissue-derived EVs [46], while CD41a identifies platelet-derived EVs [34, 47], CD45 leukocyte-derived EVs [21, 48, 49], CD31 EVs derived from the endothelium [25], and Annexin V EVs from activated compartments [50]. Total extracellular vesicle counts, as well as those of each identified subtype, were evaluated as previously reported [29, 34]. As shown in Figure S1 panel A, no statistically significant differences were observed between the groups in terms of EV counts, except for EVs CD45 + and CD41+/Annexin V + that were significantly lower and higher, respectively, in T1DM + individuals compared to healthy subjects (Figure S1 panel A) [51]. Furthermore, diameter and size distribution of each EV subtypes were measured by NTA. EVs derived from PB of T1DM+, T1DM- and HC subjects showed an average diameter of 142.9 nm, 138.5 nm and 133.3 nm, respectively, not statistically significant different in the three clinical groups (Figure S1 panel B). In this context, to study the possible diagnostic involvement of EVs in the processes associated with IR, a total of 2.9 × 10^6^ intact EVs (LCD+/phalloidin- events) were isolated and separated by instrumental cell sorting, as already reported [34, 41] from whole blood of T1DM + and T1DM- children and, then, pooled for proteomics purposes, as shown in the workflow of Fig. 1. EV protein profile of both T1DM + and T1DM- subjects was compared and normalized to that of HC using the same number of purified EVs, as already published by our group [23, 34, 41, 52].

The intensity-based absolute quantification (iBAQ) of MaxQuant was used as quantification parameter for protein expression. The first comparison (T1DM+/HC) revealed 70 and 74 proteins for T1DM + and HC EVs, respectively, with the 28.6% of proteins in common. Instead, in the second comparison (T1DM-/HC) 91 proteins were quantified for T1DM- EVs and 78 for HC EVs highlighting more common proteins equal to the 48.7%. Meanwhile, 29 (22.1%) proteins were quantified in both pathological conditions, pointing out that EVs are packaged with distinct and specific proteins depending on the presence of IR. Venn diagrams of Figure S2 showed all quantified proteins in the two performed comparisons, as listed in Supplementary Table S3, sheets “quantification”, in which a ratio of 100 indicates the exclusive quantification only in T1DM + or T1DM- EVs, depending on the performed comparison; instead a protein quantified as exclusive in HC EVs is indicated by 0.01 ratio.

**Fig. 2:**
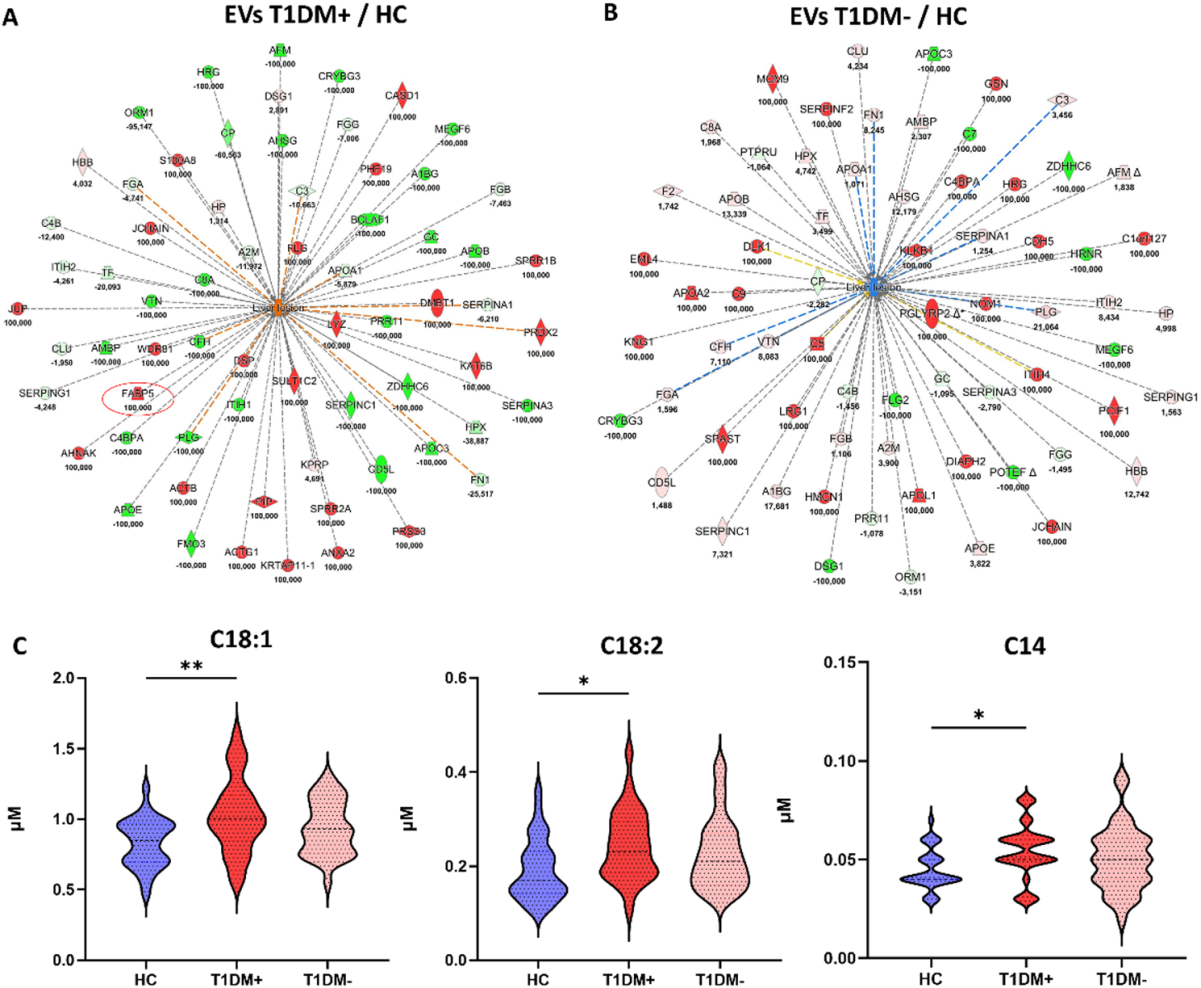
Molecular signature of T1DM paediatric patients with IR reveals specific pathways related to liver injury in contrast to T1DM without IR. **A**) Mechanistic network of *“liver lesion”* predicted by IPA software as the most significant activated downstream (z-score = 2.79) by EV proteins in the comparison T1DM + vs. HC. Fatty acid-binding protein 5 (FABP5) was quantified as unique protein in T1DM + EVs, so circled in red in the network to highlight its involvement in the activation of *“liver lesion”* in the comparison T1DM + vs. HC. It wasn’t quantified neither in T1DM- EVs nor in HC EVs. **B**) Mechanistic network of *“liver lesion”* predicted by IPA software as not significant modulated downstream effect (z-score = −1.44) by EV proteins in the comparison T1DM- vs. HC. Blue and orange shapes and lines indicate the predicted activation and inhibition, respectively. Yellow lines indicate the inconsistent finding between the molecular target and the downstream; grey lines symbolize the effect not predicted. Instead, red and green shapes represent increased or decreased measurements of quantified proteins, respectively, whose fold change value is reported under each protein. Color key and symbols for IPA interpretation are reported in Supplementary Figure S4. **C**) DBS levels of long-chain acylcarnitines in T1DM patients with and without IR compared to healthy subjects. IR was calculated following the mathematics model of estimated Glucose Disposal Rate (eGDR). Data are visualized as violin plots where the mean is indicated by dotted black lines and the polygon shapes represents the density trace of ACs in term of concentrations (µM). ** means p-value < 0.01, * means p-value < 0.05 obtained by Tukey’s multiple comparisons test

Protein ratios for both comparisons were upload on Ingenuity Pathway Analysis (IPA tool) for Core Analyses. Downstream effects analysis revealed that EV protein cargo of T1DM paediatric patients was able to trigger pathological functions related to *“liver lesion”*; in particular, such function was the most significant activated downstream in T1DM + EVs examined in relation to HC EVs (Fig. 2A, z-score = 2.79; p-value = 1.38 × 10−8). On the other hand, the same pathological function was slightly inhibited, but not significantly modulated, by EV proteins of T1DM- subjects compared to HC (Fig. 2B, z-score =−1.44; p-value = 1.33 × 10^−8^). The complete list of downstream effects was reported in Supplementary Table S4, sheets “DS T1DM+” and “DS T1DM-”.

Mechanistic network of Fig. 2A showed that the strong activation of *“liver lesion”* was exploited by 67 T1DM + EV proteins, between them fatty acid-binding protein 5 (FABP5) was quantified as exclusive protein in T1DM + EVs (fold change = 100), and not in T1DM- EVs, as highlighted by red circle in the network of Fig. 2A. FABPs are a family of small cytoplasmic proteins that bind long-chain fatty acids and other hydrophobic ligands [53] and, simultaneously, polymorphisms in *FABP5* gene are associated with T2DM. Therefore, we measured the levels of long-chain acyl-carnitines in DBS samples collected by spotting PB of T1DM+ (*n* = 29), T1DM- (*n* = 35) and HC subjects. In particular, as shown in Fig. 2C, unsaturated long-chain oleoylcarnitine (C18:1) and linoleylcarnitine (C18:2), and satured myristoylcarnitine (C14) levels were significantly higher in T1DM + patients respect to HCs (p-value = 0.0018 for C18:1; p-value = 0.015 for C18:2; p-value = 0.022 for C14). Other long-chain ACs, such as palmitoylcarnitine (C16), stearoylcarnitine (C18) and palmitoleoylcarnitine (C16:1) were not significantly different between HC and T1DM+/- subjects (Supplementary Figure S3). Notably, although not significant, a downward trend was observed in T1DM- DBS for C18:1 respect to T1DM+, whose serum levels increased with the progression of liver fibrosis and were higher in patients with hepatocellular carcinoma (HCC) [54]. These data corroborate our EV proteomics results, suggesting that EV protein cargo is functionalized to trigger specific messages linked to liver lesion and injury, from steatosis to cirrhosis and/or HCC, in T1DM patients with IR since it is implicated in the pathogenesis of MAFLD [54].

### Suppression of fatty acid metabolism via STAT3 Inhibition through extracellular vesicles in diabetes type 1 with insulin resistance

**Fig. 3:**
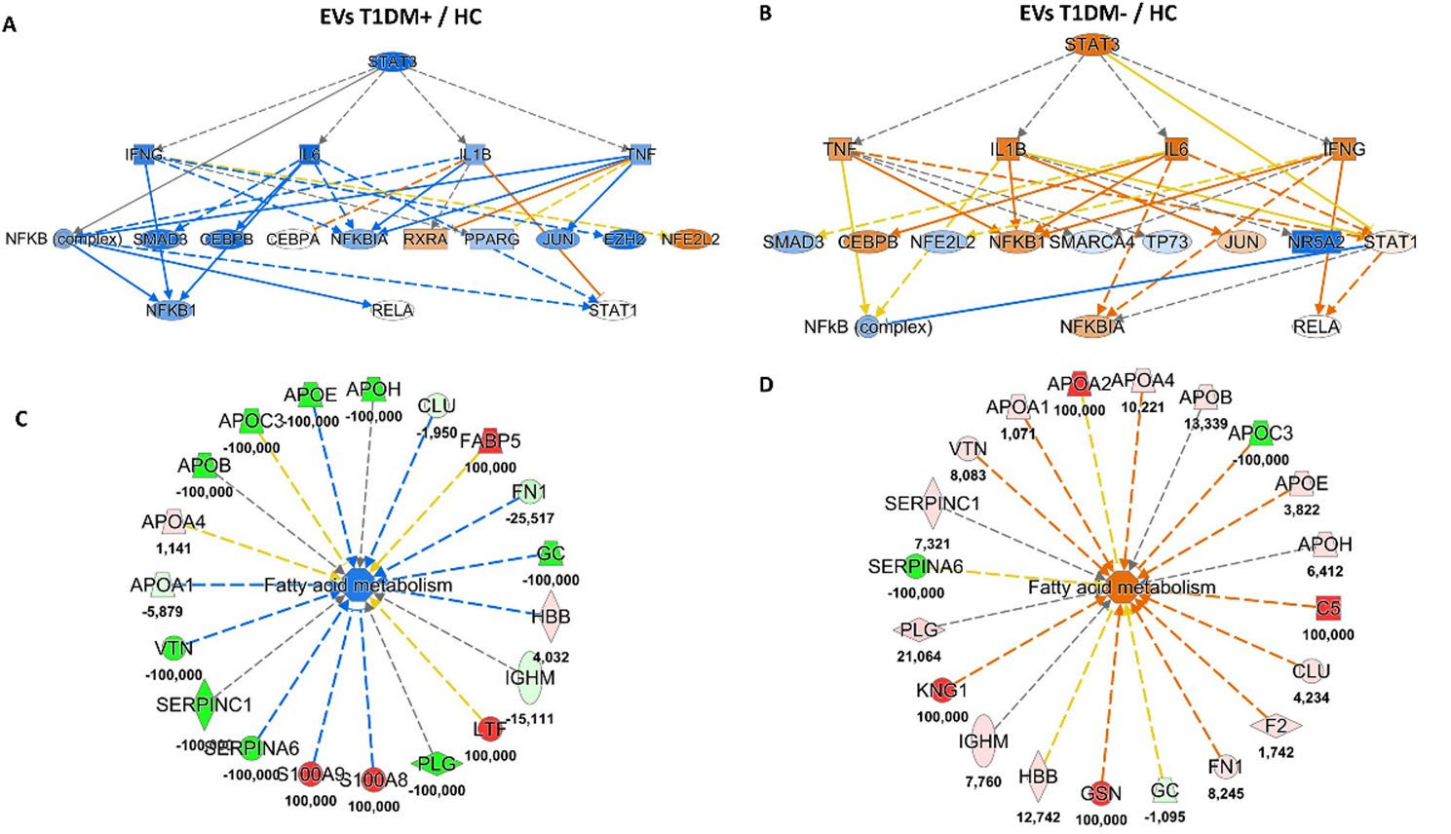
Functional comparison analysis by IPA tool reveals a suppression of fatty acid metabolism through *STAT3* deactivation mediated by EVs in T1DM paediatric patients with IR. **A**) Upstream regulator network of signal transducer and activator of transcription 3 *(STAT3)* predicted as the most inhibited upstream (z-score = −2.66) by EV proteins of T1DM + compared to HC EVs. **B**) On the other hand, *STAT3* gene was significantly activated (z-score = 2.10) in the comparison T1DM-/HC. **C)** Downstream effects of *“fatty acid metabolism”* was slightly downregulated, but not significantly, (z-score = −1.54) through EV proteins in T1DM + patients vs. healthy volunteers. **D**) Instead, the same biofunction was significantly upregulated (z-score = 2.12) in the comparison T1DM-/HC. Blue and orange shapes and lines indicate the predicted activation and inhibition, respectively. Yellow lines indicate the inconsistent finding between the molecular target and its downstream molecules; grey lines symbolize the effect not predicted. Color key and symbols for IPA interpretation are reported in Supplementary Figure S4

Building on the hypothesis of liver damage and the potential effects of long-chain ACs species in disparate pathophysiology of inflammation and IR in T1DM, we investigated the role of EVs in mediating these biofunctions, starting from the upstream regulators that could have been modulated by the origin cells. As a matter of fact, as shown in the network of Fig. 3A the most significant inhibited upstream in T1DM + EVs respect to HC EVs was signal transducer and activator of transcription 3 *(STAT3)* with a z-score of −2.66 (p-value = 7.64 × 10^−10^). Conversely, in the comparison T1DM-/HC *STAT3* was predicted as significant activated upstream (z-score = 2.10, p-value = 6.16 × 10^−9^), as reported in Fig. 3B. Accordingly, our proteomics data showed that this modulation at origin cells level was exploited by EVs that were well packaged to deliver information related to *“fatty acid metabolism”*, whose function was slightly reduced in T1DM + EVs (z-score = −1.54, p-value = 9.27 × 10^−9^, Fig. 3C), but it was predicted significantly activated in T1DM- EVs (z-score = 2.12, p-value = 2.10 × 10^−9^, Fig. 3D) compared to HC ones. Together with a higher expression of C18:1 in DBS samples of T1DM + patients, these data demonstrated that could be evident a suppression of fatty acid β-oxidation via *STAT3* activation mediated by EVs that might reflect the toxic function of *“liver lesion”* highlighted in the previous paragraph. It was demonstrated that C18:1 contributes to hepatocarcinogenesis through *STAT3* activation [54]. The phosphorylated *STAT3*, in fact, plays a key role in inducing the acute phase response, promoting hepatocyte survival and liver regeneration [55].

The complete list of upstream regulators was reported in Supplementary Table S4, sheets “US T1DM+” and “US T1DM-”.

**Fig. 4:**
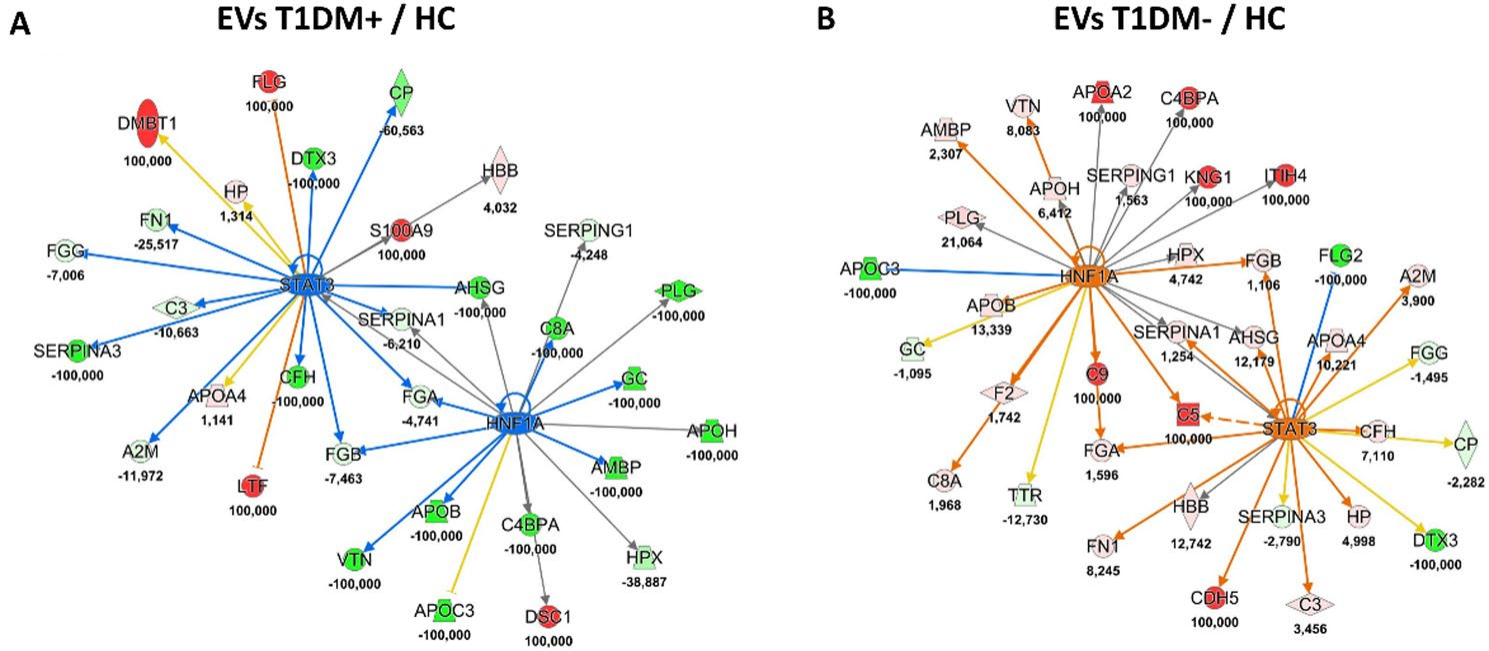
Interactive network of *STAT3* and *HNF1A* as upstream regulators predicted by EV proteins of T1MD paediatric patients. **A**) Hepatocyte nuclear factor 1 A *(HNF1A)* and signal transducer and activator of transcription 3 *(STAT3)* and*)* were two of the most inhibited upstreams in comparison EVs T1DM+/HC (z-score = −2.16 for *HNF1A* and − 2.66 for *STAT3*). **B**) On the contrary, they were significantly activated upstream regulators in the comparison EVs T1DM-/HC (z-score = 2.03 for *HNF1A* and 2.10 for *STAT3*). Lines represent the predicted direct (solid lines) relationship among the genes and quantified proteins. Color of the lines represent the inhibition (blue) or the activation (orange), inconsistent (yellow), and not prediction (grey). Color key and symbols for IPA interpretation are reported in Supplementary Figure S4

**Fig. 5:**
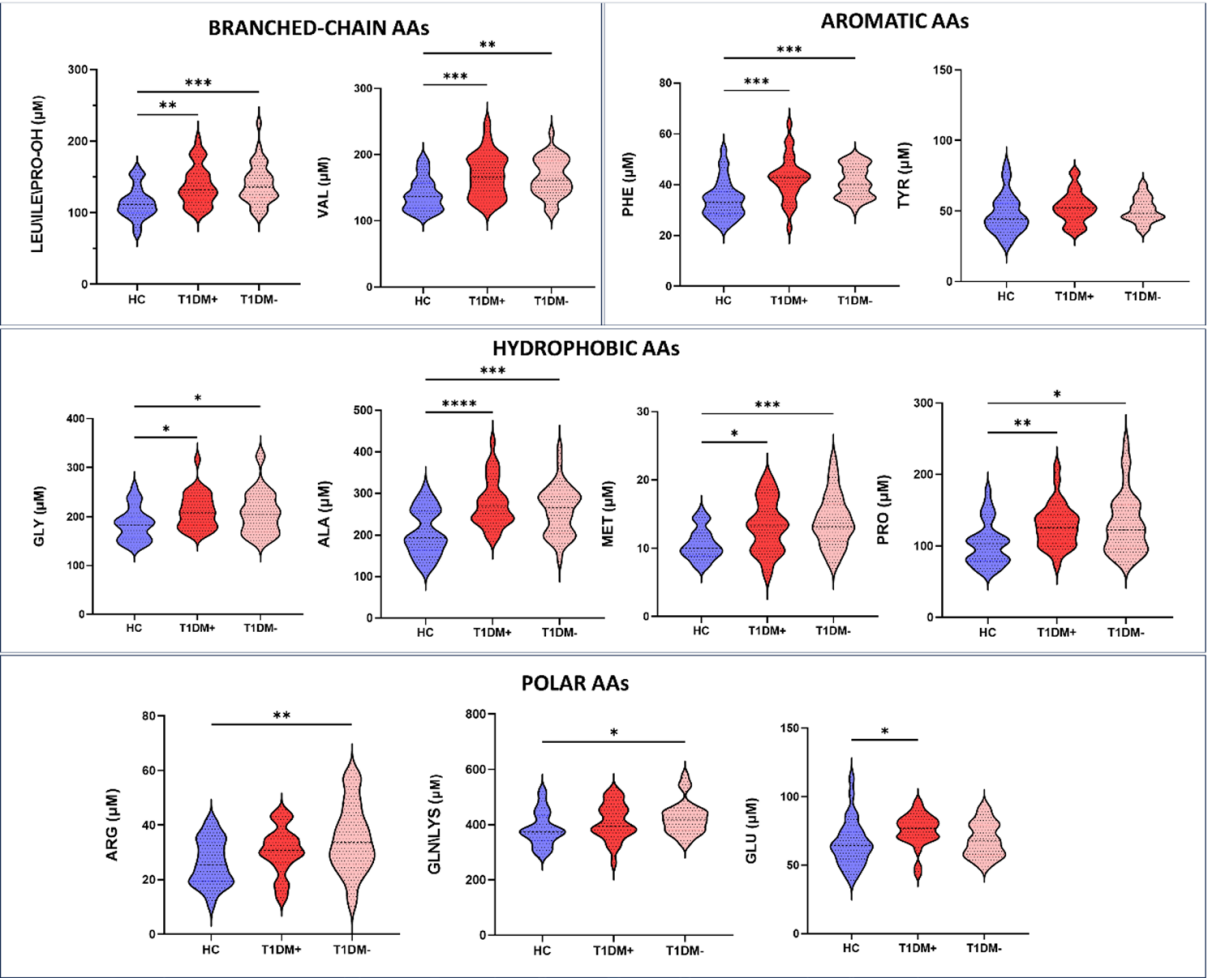
Distribution of AAs evaluated on DBS samples con healthy volunteers and T1DM patients with (T1DM+) and without (T1DM-) insulin resistance. Data are visualized as violin plots where the mean is indicated by dotted black lines and the polygon shapes represents the density trace of AAs in term of concentrations (µM). IR was calculated following the mathematics model of estimated Glucose Disposal Rate (eGDR). **** means p-value < 0.0001, *** means p-value < 0.001, ** means p-value < 0.01, * means p-value < 0.05 obtained by Tukey’s multiple comparisons test for normally distribution or by Kruskal-Wallis test with Dunn’s multiple comparisons test for not-normally distribution. The analytes reported with multiple nomenclatures are isobar molecules and cannot be distinguished with FIA-MS/MS

In addition, delving deeper into the molecular information packaged in T1DM EVs, our proteomics data pointed out how EV proteins could predict significant modulation of *HNF1A* and *STAT3* genes. In particular, Fig. 4A showed the interactive network of *HNF1A* (z-score = −2.16, p-value = 2.83 × 10^−10^) and *STAT3* (z-score = −2.66, p-value = 7.64 × 10^−10^) as significant inhibited upstream regulators by T1DM + EVs compared to HC ones. Conversely, in the comparison T1DM-/HC EV proteins were able to predict a significant activation of the same genes: *HNF1A* (z-score = 2.03, p-value = 1.48 × 10^−16^) and *STAT3* (z-score = 2.10, p-value = 6.16 × 10^−9^), as displayed in Fig. 4B. The simultaneous modulation of *HNF1A* and *STAT3* as regulatory factors supported the suppression of fatty acid metabolism in T1DM young people with IR through EVs, potentially leading to liver damage, since *HNF1A* plays an important role in maintaining liver lipid homeostasis [56]. At the same time, these data were corroborated by circulating metabolites evaluated by FIA-MS/MS on DBS samples of the same subjects. *HNF1A* gene has a well-documented regulation of *PAH*, the enzyme that converts PHE in TYR; in fact, increased levels of urinary PHE were detected in HNF1A-null mouse models [57]. Our data are in agreement with these findings because PHE was higher in T1DM DBS samples compared to HC and, even if, no significant differences were found between T1DM + and T1DM- patients, a slight increase of PHE was revealed in T1DM samples with IR or “double diabetes” (see Fig. 5 and Table S3), as some single nucleotide polymorphisms of *HNF1A* gene increase the susceptibility to T2DM or gestational diabetes mellitus (GDM) [58].

### DBS amminoacid species in children and adolescents living with double diabetes

Although many metabolomics studies have been conducted on the development of insulin resistance (IR) and T2DM in adults, the studies in T1DM and paediatric diabetes are more limited. This study aimed to explore the metabolic signature in “double diabetes” in terms of AAs and ACs by FIA-MS/MS. In particular, we analyzed AAs levels on DBS samples collected by spotting PB of 29 T1DM+, 35 T1DM-, and 31 HC subjects, onto filter paper. The metabolomics profile of samples showed a significant modulation in T1DM in respect to HC in term of branched-chain amminoacids (BCAAs), hydrophobic amminoacids, and positive polar amminoacids levels, as reported in violin plots of Fig. 5. Considering BCAAs, we found a significantly increasing of Leucine/Isoleucine (LEU\ILE\PRO-OH) and Valine (VAL) in T1DM paediatric patients respect to HC. The same trend was observed for hydrophobic amminoacids, as glycine (GLY), alanine (ALA), metionine (MET) and Proline (PRO), as well as for positive polar AAs as, arginine (ARG), glutamine\lysine and (GLN\LYS).

Considering Aromatic AAs as Phenilalanine (PHE) and Tyrosine (TYR), we found a significantly higher level of PHE in T1DM DBS samples vs. HC, while no difference was found in TYR levels. Moreover, even if there were no differences between T1DM + and T1DM- in Aromatics AAs levels, PHE was slightly increased in DBS samples of T1DM + children compared to T1DM- ones. Supplementary Table S3 summarized the FIA-MS/MS results in terms of AAs and ACs concentrations.

### Clinical characteristics models integrating clinical data and metabolites for T1DM with and without insulin resistance

**Fig. 6:**
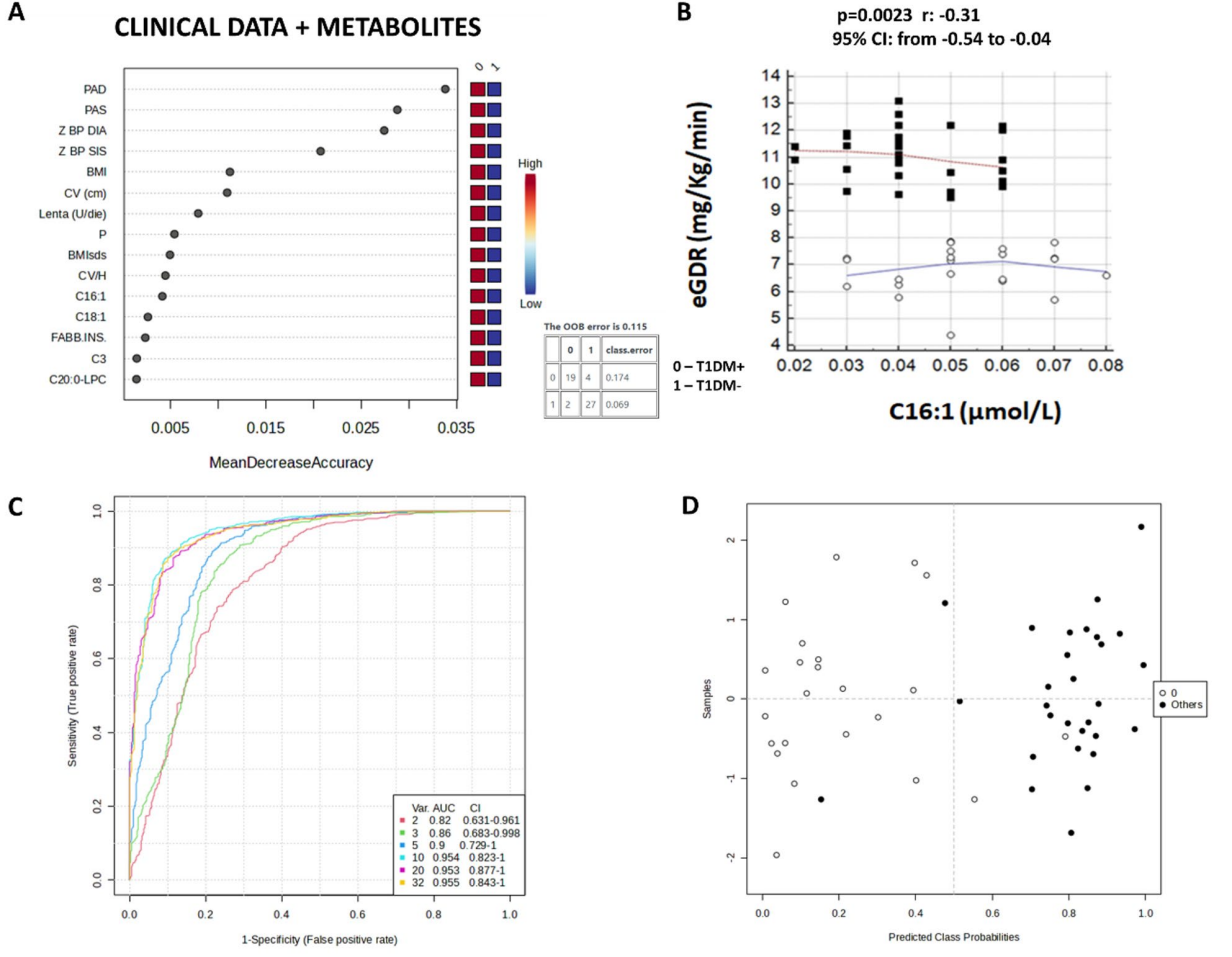
Clinical characteristics model for discriminate children T1DM with and without insulin resistance. **(A)** Independent random forest models for combination of metabolite and clinical data. **(B)** Significant correlation between eGDR and long-chain acyl-carnitine C16:1. **(C)** Cumulative ROC curves 2, 3, 5, 10, 20 and 32 features, respectively. Area under the curve (AUC) and confidence interval (CI) values are reported. **(D)** Confusion matrix after cross-validation of the cumulative ROC curve obtained with 32 features, the best model to distinguish T1DM paediatric patients with and without IR. The T1DM + and T1DM- samples were displayed as black and white dots, respectively

Our proteomics data have unravelled that blood EVs in T1DM paediatric patients were well packaged in order to deliver specific information related to IR according to fatty acid metabolism and possible liver injury. These data were validated by measuring circulating ACs levels in DBS obtained from whole blood of the same patients. Since in the paediatric T1DM population the eGDR value have provided contrasting information, we tried to integrate clinical features with metabolites levels with machine learning algorithms, in particular random forest model. The random forest model that included both metabolite values and clinical factors had an out-of-bag error of 0.115% (Fig. 6A) demonstrating that long-chain acyl-carnitines palmitoleoylcarnitine (C16:1) and oleoylcarnitine (C18:1) were the metabolites that better discriminate children with T1DM + from T1DM-. It’s important to underline that in every random forest models palmitoleoylcarnitine (C16:1) was the first significant feature used for building the models; in fact, C16:1 was significantly higher in T1DM+ (p-value = 0.04 at T-test) and negatively correlated with eGDR values which were used to classify young people managing T1DM (p-value = 0.0023, Spearman’s rho: −0.314, 95% CI from − 0.541 to −0.0451, Fig. 6B). The area under the curve (AUC) for this combined model was 0.954, as shown by yellow curve of the ROC curve of Fig. 6C. In this regard, a ROC curve-based multivariate analysis was carried out for all clinical factors (without eGDR) and C16:1 and C18:1 values with linear Support Vector Machine (SVM) classification method. ROC analyses were obtained with different numbers of features (2, 3, 5, 10, 20, 32) and their AUC were able to distinguish between two diagnostic groups with and without IR (Fig. 6C). The best model, depicted in the best confusion matrix of Fig. 6D, was based on 32 features which have included C16:1 and C18:1. In particular, 2/23 T1DM+ (labelled 0) and 2/29 T1DM- (labelled 1) were misclassified, with an error of prediction of 8.70% and 6.90%, respectively. These misclassifications cannot be explained based on the information available in the current study.

## Discussion

This study provides new insights into the molecular cross-talk underlying IR in paediatric patients with T1DM by integrating EV-based proteomics and targeted metabolomics from DBS. We characterized the metabolic signature of 64 children and adolescents with T1DM dividing them in with (T1DM+) and without (T1DM-) IR according to eGDR score (mg/Kg/min), validated for T1DM adult patients, but not specifically for children. eGDR levels were associated with increased subsequent risk of both micro- and macrovascular complications [58]. The rate of overweight and obesity was similar to other groups of children with T1DM reported [59, 60]. About 73% of the study population was considered pubertal, but with eGDR stratification, pubertal patients were equally distributed between the two groups. This is an important finding considering that insulin sensitivity decreases physiologically by 30% in mid puberty favouring protein synthesis and growth [61]. In this series, 28.8% of patients had prehypertension and one patient had systolic blood pressure over the 95th percentile for age, sex and height percentile, according to criteria of the National High Blood Pressure Education Program Working Group [62]. Although previous paediatric studies only assessed hypertensive children and adolescents in calculating the score, subjects with prehypertension were considered in calculating eGDR, because management guidelines suggest a lifestyle-change intervention even at this stage. It can be said that the phenotypic and metabolic characteristics of patients with T1DM and IR are getting closer to those of subjects with MetS or T2DM; for this reason, considering the lack of studies on paediatric population with T1DM and IR, we investigated the metabolism of this underinvestigated cohort of patients in relation to metabolomics signature and EV protein cargo evaluating them as functionalized shuttles towards b-cells and mesenchymal stem cells, skeletal muscle cells, and adipocytes [17].In recent years, studying molecular cargo of EVs isolated from human biological fluids is a very challenging task for researchers, especially in relation to EV protocol isolation suitable for proteomics purposes. Circulating EVs have been found altered in T2DM body fluids of patients with metabolic dysfunctions and complications [63], but their role in “double diabetes” patients is still completely underinvestigated. In this complex context, our research group has developed an innovative FACS-Proteomics strategy for studying protein cargo of EVs directly purified from untouched biofluids by instrumental cell sorting [21, 32, 34, 41]. The combination of the high powerful capabilities of FACS and high-resolution mass spectrometry allowed us to better characterize the pathophysiology of T1DM and IR, providing a unique metabolic snapshot in such a complex context involving diabetes-related complications especially linked to obesity and IR in paediatric subjects. In particular, we applied our patented method to purify total circulating EV cargo from whole blood of T1DM + and T1DM- patients to compare their own EV protein fingerprint with control one. We identified and count circulating EVs, as well as adipocyte, leukocyte, endothelial and platelet-derived EVs, also considering their positivity to Annexin V. At first, no significant differences in terms of EV size distribution and counts between T1DM and healthy population. Simultaneously, leukocytes-derived EVs and platelets-derived EVs positive to Annexin V were significantly lower and higher, respectively, in T1DM + individuals compared to HCs in line with previous studies where adults with MetS showed reduced levels of CD45 + EVs [51] and higher platelet-derived EVs levels were found in T2DM subjects with IR as marker for vascular dysfunction [64] and liver pathophysiology [65], as we will discuss later. Meanwhile, our encouraging proteomics data highlighted as T1DM EVs are packaged with a well-defined protein set according to the presence or not of IR. As a matter of fact, on the one hand T1DM + EVs are able to vehiculate pathological messages related to liver lesion (Fig. 2) and fatty acid metabolism (Fig. 3) unravelling not only specific EV proteins, such as FABP5, involved in obesity and obesity-induced IR [66], but also the functional and active role the EVs have in diabetes-related complications associated to IR, as demonstrated by higher levels of unsaturated and saturated long-chain ACs (C18:1, C18:2 and C14) found in DBS of T1DM + children patients respect to T1DM- ones. Among the most relevant findings of our proteomics analysis, the identification of FABP5 as an exclusive component of EVs from T1DM + subjects represents a notable molecular hallmark. FABPs are a family of intracellular lipid chaperones that bind long-chain fatty acids and other hydrophobic molecules, playing a central role in fatty acid uptake, transport and metabolism [66]. Genetic variants in the *FABP5* gene have been associated with increased susceptibility to T2DM. In particular, both FABP4 and FABP5 have emerged as key contributors to obesity-induced IR: their dual silencing in white adipose tissue significantly reduced inflammation and improved insulin sensitivity in preclinical models [66]. Our finding reinforced the hypothesis that T1DM + EVs carry relevant information, potentially contributing to the onset of complications related to IR and liver injury, such as MAFLD, also supporting the concept that the metabolic phenotype of “double diabetes” in paediatric populations shares molecular features with MetS and T2DM. These data supported our finding that T1DM + children were significantly older and had a longer disease duration than T1DM- in line with previous studies in which has been showed that IR can develop during the preclinical stages of T1DM prior to its clinical manifestation, as reported by Apostolopoulou and colleagues [67]. This clinical aspect may contribute to cumulative metabolic stress, mitochondrial abnormalities and alterations in fatty acid metabolism as demonstrated by EV proteomics profile too. In addition, the targeted metabolomic analysis of DBS revealed a distinct pattern of circulating long-chain acylcarnitines in T1DM + patients. Specifically, levels of oleoylcarnitine (C18:1), and myristoylcarnitine (C14) were significantly elevated in T1DM + children compared to HC, suggesting impaired mitochondrial fatty acid β-oxidation and a possible metabolic overload. In fact, as demonstrated by Enooku et al., serum levels of C14:1 and C18:1 increased with the progression of liver fibrosis, but only C18:1 levels resulted increased in patients with MASH compared to obese control subjects [54]. Although other long-chain acylcarnitines, such as palmotoylcarnitine (C16), stearoylcarnitine (C18) and palmitoylcarnitine (C16:1) did not reach statistical significance, a downward trend of C18:1 was observed in T1DM- subjects, further distinguishing them from the insulin-resistant phenotype. Moreover, upstream regulator analysis revealed that EVs could exploit the downregulation of fatty acid β-oxidation via *STAT3* activation highlighting a strong inhibition of *STAT3* as upstream in T1DM + EVs compared to HC in contrast to T1DM- EVs (Fig. 4). Even though there is no direct evidence that hyperinsulinemia itself adversely affects the liver, animal studies suggest that insulin is a direct cause of both hepatic steatosis and fibrosis [54]. In this regard, our “multi-omics” approach revealed that EVs T1DM + trigger functional information related to IR complications in the paediatric diabetic population, such as liver lesion and injury associated to MASH and MAFLD [54]. In fact, *STAT3* exerts a positive and beneficial effect in conditions of liver damage, steatosis, fatty liver disease, or inflammation since it ensures normal liver regeneration [55]. Previous studies showed that adults with T1DM had a higher inclination to metabolic dysfunction-associated steatotic liver disease (MASLD), as correctly identified by guidelines through hepatic steatosis index (HSI), which is a composite marker, computed by combining serum transaminase levels, body mass index, gender and presence of diabetes recently validated in adult patients with T1DM [68, 69]. Instead, serum liver enzyme levels in T1DM children and adolescents could be normal and not satisfied these guidelines because age and disease duration are critical determining factors. Paediatric and adolescent populations generally show lower MASLD prevalence compared to adult cohorts due to shorter disease duration and lower cumulative metabolic burden. In this context, serum aminotransferase levels may underestimate steatosis in asymptomatic patients, such as paediatric T1DM population [68, 69]. However, their evaluation could be useful in the following steps of the study to further assess links between EVs protein cargo, IR, and early hepatic metabolic alterations in paediatric T1DM. Moreover, *HNF1A* gene was significantly downregulated as upstream by EV protein dataset of T1DM + patients in contrast to T1DM-. Since it has a key role in maintaining liver lipid homeostasis, we demonstrated once again not only that T1DM + EVs may trigger functional information related to liver injury. Interestingly, adipocytes release EVs containing bioactive cargos (miRNAs, adipokines, lipids) impair insulin signalling in hepatocytes. It has been demonstrated that EVs from human adipose tissue explants inhibited insulin-induced Akt phosphorylation in hepatocytes and correlated with elevated liver enzymes [70]. Furthermore, platelet-derived EVs have been implicated in liver pathophysiology (including acute injury, ischemia/reperfusion, fibrosis) [65], suggesting a mechanistic route for platelet EVs to impact liver lesion progression. EVs derived from endothelium or leukocytes are recognized mediators of vascular and inflammatory signalling and may promote IR and vascular dysfunction, impacting metabolic organs including liver via endocrine signalling [64]. Therefore, the presence of adipose-, platelet-, leukocyte- or endothelial-derived EVs suggests a composite EV milieu, and the liver may be both target and source in a dynamic inter-organ dialogue of EVs. Furthermore, the different identified EV subtypes may provide phenotypic support for such multi-tissue origins and crosstalk, possibly contributing to the “liver lesion” signature identified by proteomics. In addition to the metabolic alterations observed in T1DM + patients, our targeted metabolomics analysis of AAs provided further insights into the systemic metabolic dysregulation associated with insulin resistance in this paediatric population. Notably, levels of PHE were significantly higher in T1DM + DBS samples compared to HCs; while TYR levels remained unchanged. Although no statistically significant difference in PHE levels was observed between T1DM + and T1DM- subgroups, a mild upward trend was evident in the insulin-resistant cohort (Fig. 5, Table S3). This pattern supplemented the proteomics evidence of *HNF1A* as downregulated upstream by T1DM + EV proteins, as it is known to transcriptionally regulate phenylalanine hydroxylase (PAH), the enzyme that coverts PHE to TYR [57]. Certain *HNF1A* gene variants have been linked to increased risk of T2DM and GDM [58]. Together, these amminoacids alterations, further support the concept that paediatric patients with “double diabetes” exhibit a distinct and early metabolic phenotype characterized by hepatic vulnerability and IR. Moreover, compared to HCs, T1DM children and adolescents exhibited significantly elevated levels of BCAAs - including leucine/isoleucine/proline-OH and valine - as well as hydrophobic AAs (glycine, alanine, methionine, and proline) and positively charged polar AAs (arginine, glutamine/lysine), as shown in Fig. 5. These findings are consistent with existing evidence linking increased BCAAs to insulin resistance and altered mitochondrial metabolism [71]. Finally, considering the limitations of the eGDR parameter, already discussed, in characterizing IR in children with T1DM, we explored whether integrating clinical data with circulating metabolite levels could improve this classification. Of all the variables considered, C16:1 and C18:1 resulted the most informative in distinguishing T1DM + from T1DM- patients. In particular, C16:1 consistently ranked as the leading feature in the integrated model and was significantly elevated in the T1DM + population. It also showed a significant negative correlation with eGDR, further strengthening its potential role as a biomarker of IR. Although the modest sample size represents a limitation of our study and may affect the statistical power of our findings, its exploratory nature provided valuable preliminary insights into this underinvestigated paediatric T1DM cohort, as a magnifying glass we were able to unravel both novel putative metabolic biomarkers and a specific EV-mediated molecular fingerprinting of IR in double paediatric patients. In this regard, future studies based on our molecular findings could pave the way for a better comprehension of IR mechanisms in a multifactorial disease such as T1DM, using independent case cohorts in which not only clinical outcomes but also the different treatments and lifestyles have to take into account.

## Conclusions

Our results reinforce the idea that combining targeted metabolomics with basic clinical information may offer a more reliable and biologically grounded method to identify IR in T1DM children. In particular, C16:1 and C18:1 emerge not only as indicators of altered fatty acid metabolism, but also as useful discriminative features for identifying children at higher metabolic risk. This integrative approach could support a more personalized disease monitoring and may eventually complement the use of eGDR in clinical practice.

## Supplementary Information

Supplementary Material 1

Supplementary Material 2

Supplementary Material 3

Supplementary Material 4

Supplementary Material 5

## Data Availability

No datasets were generated or analysed during the current study.

## Abbreviations

T1DM: Type 1 diabetes mellitus
T2DM: Type 2 diabetes mellitus
IR: Insulin resistance
T1DM+: Patients with type 1 diabetes mellitus with insulin resistance
T1DM-: Patients with type 1 diabetes mellitus without insulin resistance
eGDR: Estimated Glucose Disposal Rate
MetS: Metabolic syndrome
MAFLD: Metabolic associated fatty liver disease (MASH metabolic associated steatohepatitis)
MASLD: Metabolic dysfunction-associated steatotic liver disease
HbA1c: Glycosilated hemoglobin
EVs: Extracellular vesicles
LCD: Lipophilic cation dye
LC-MS/MS: Liquid chromatography tandem mass spectrometry
FIA-MS/MS: Flow injection analysis-tandem mass spectrometry
DBS: Dried blood spot
AAs: Amminoacids
ACs: Acylcarnitines
BCAAs: Branched-chain amminoacids
iBAQ: Intensity-based absolute quantification
IPA: Ingenuity Pathway Analysis
NTA: Nanoparticle tracking analysis
