# Supplementary material for: Molecular cross-talk via extracellular vesicles for the characterization of young subjects with type 1 diabetes unravels new potential markers of insulin resistance and double diabetes
Source: Diabetol Metab Syndr. 2025 Dec 10;18:16. doi: 10.1186/s13098-025-02042-7 (PMC12801652; doi:10.1186/s13098-025-02042-7)
Supplement: Supplementary file 3 — Supplementary Material 3 [file 13098_2025_2042_MOESM3_ESM.docx]

Supplementary Materials of:

Molecular cross-talk via Extracellular Vesicles for the characterization of type I diabetes patients unravels new potential markers of insulin resistance and double diabetes

Maria Concetta Cufaro ^1,2^, Ilaria Cicalini ^2^, Paola Irma Guidone ^3^, Paola Lanuti ^2,4^, Francesca D’Ascanio ^2,4,5^, Maria Alessandra Saltarelli ^6^, Lorenza Sacrini ^6^, Domenico De Bellis ^2,4^, Gessica Di Carlo ^2,7^, Luca Natale ^1,2^, Damiana Pieragostino ^1,2^, Piero Del Boccio ^2,7^, Claudia Rossi ^2,7^ * and Stefano Tumini ^6^

1 Department of Innovative Technologies in Medicine and Dentistry, G. d’Annunzio University of Chieti-Pescara, 66100 Chieti, Italy

2 Center for Advanced Studies and Technology (CAST), G. d’Annunzio University of Chieti-Pescara, 66100 Chieti, Italy

3 UOSVD Paediatrics, Della Murgia Fabio Perinei Hospital, 70022 Altamura (BA), Italy

4 Department of Medicine and Aging Sciences, G. d’Annunzio University of Chieti-Pescara, 66100 Chieti, Italy

5 Department of Humanities, Law and Economics, Leonardo da Vinci University, 66010 Torrevecchia Teatina (CH), Italy

6 Department of Maternal and Child Health, UOSD Regional Center of Paediatric Diabetology, Chieti Hospital, 66100 Chieti, Italy

7 Department of Science, G. d’Annunzio University of Chieti-Pescara, 66100 Chieti, Italy

***** Correspondence: Claudia Rossi, [claudia.rossi@unich.it](mailto:claudia.rossi@unich.it), +39 0871 541329

**Figure S1.** Venn Diagrams of EV quantified proteins in the two comparisons.


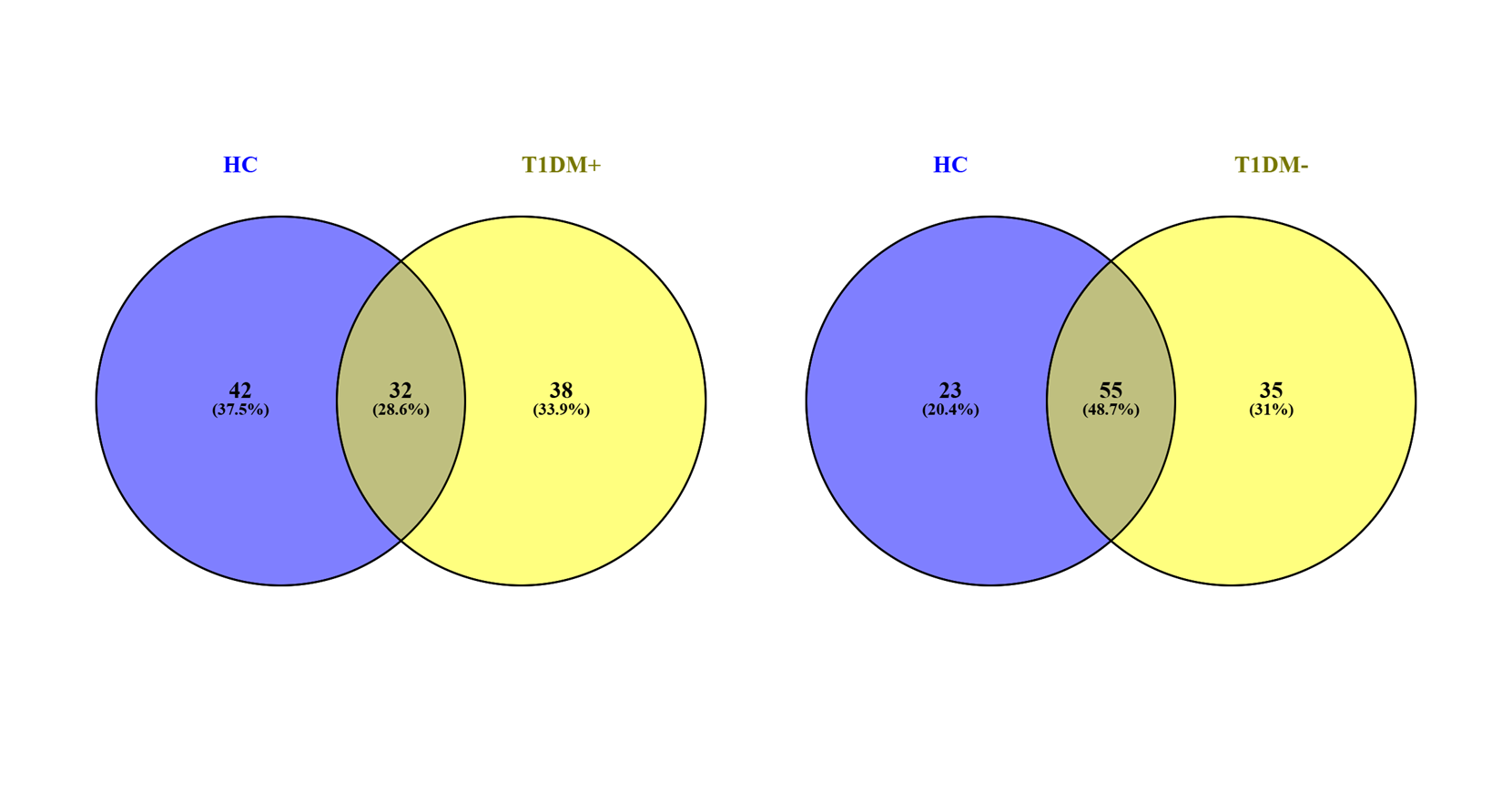


**Figure S2.** Long-chain ACs that were not significantly different between the clinical groups.


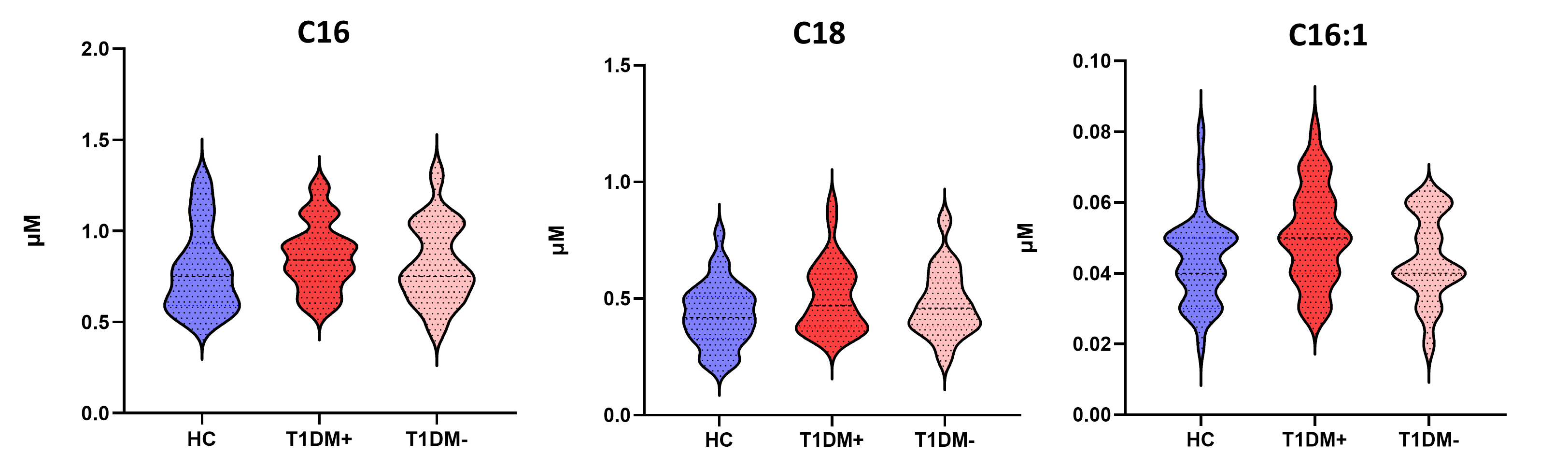


**Figure S3.** IPA legend interpretation. The ﬁgure shows the colour and shape key for IPA networks.

**
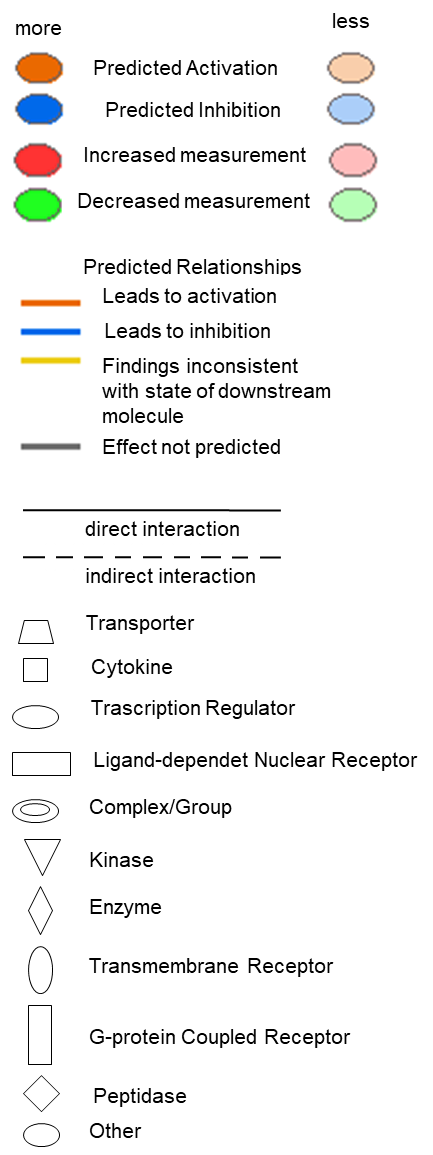
**

**Table S1.** General features of the paediatric population involved in the study.

| **Subjects** | **total = 52** |
| --- | --- |
| Males (%) | 53.9 |
| Median age (years) | 13.7 (r: 10.9 – 15.8) |
| Age > 11 years (%) | 73.1 |
| Years from diagnosis (mean + sd) | 5.7 + 3.6 |
| Height sds (mean + sd) | 0.73 + 1.26 |
| Weight sds | 0.9 + 0.7 |
| BMI sds | 1.64 + 1.13 |
| BMI > 25 Kg/m^2^ (%) | 25 (n=13) |
| WHtR > 0,50 | 28.8 (n=15) |
| SBP Z score | 0.17 (r: -0.89 – 0.87) |
| DBP Z score | 0.38 (r:-0.4-1.31) |
| HbA1c > 7.5% (%) | 34 (n=18) |
| HbA1c (%) | 7.2 + 1.1 |
| Glycemic means(last 3 months) | 163 + 32 |
| SD of glycemic mean | 73.5 +19.5 |
| % CV (SD/M%) | 44 + 7 |
| Median eGDR (mg/Kg/min) | 9.6 (r: 7.2 – 11.1) |
| ISF (mg/dl) | 50 (r: 35 - 80) |
| Insulin requirement (U/Kg/day) | 0.95 + 0,34 |
| Basal dose/day (U/day) | 20.5 (r: 14 – 30.2) |
| Basal dose/Kg (U/Kg) | 0.38 + 0.15 |
| I:CHO (breakfast) (gr) | 8.5 (r: 7 - 12) |
| I:CHO (lunch) (gr) | 10 (r: 7 – 14.2) |
| I:CHO (dinner) (gr) | 10 (r: 7 – 12.5) |
| Familiar history for T2DM (%) | 63 (n = 33) |
| > 2 relatives with T2DM (%) | 28 (n = 15) |
| LDL> 100 mg/dl (%) | 38 (n = 20) |
| HDL < 40 mg/dl (%) | 7.7 (n = 4) |
| Triglicerides > 150 mg/dl (%) | 0.02 (n = 1) |
| CSII (%) | 17 (n = 9) |
| Glargine (%) | 28 (n = 15) |
| Degludec (%) | 53 (n =28) |
|  |  |

**Table S2.** List of median and standard deviation (SD) of DBS aminoacids (AA), free carnitine (C0), acylcarnitines (ACs), succinylacetone (SA), nucleosides and lysophospholipids analyzed.

| **Analyte** | **Median T1DM+** | **SD** | **Median T1DM-** | **SD** | **Median HC** | **SD** |
| --- | --- | --- | --- | --- | --- | --- |
| ALA | 269.78 | 57.76 | 266.07 | 59.16 | 193.65 | 55.25 |
| ARG | 30.69 | 8.62 | 33.64 | 12.37 | 25.64 | 187.28 |
| CIT | 21.47 | 4.64 | 23.24 | 5.11 | 20.36 | 6.26 |
| GLN\LYS | 394.69 | 64.88 | 417.17 | 59.84 | 373.08 | 61.41 |
| GLU | 76.67 | 13.81 | 67.64 | 12.50 | 64.3 | 16.71 |
| GLY | 208.96 | 35.76 | 205.24 | 43.81 | 182.48 | 33.66 |
| LEU\ILE\PRO-OH | 131.90 | 27.78 | 136.83 | 42.47 | 111.61 | 22.77 |
| MET | 13.47 | 5.09 | 13.84 | 5.44 | 10.07 | 2.93 |
| ORN | 63.71 | 15.44 | 62.86 | 17.81 | 57.34 | 115.18 |
| PHE | 42.82 | 8.77 | 41.07 | 9.76 | 32.98 | 7.50 |
| PRO | 125.41 | 30.26 | 129.66 | 58.67 | 103.05 | 29.81 |
| SA | 0.20 | 0.03 | 0.18 | 0.04 | 0.18 | 0.05 |
| TYR | 52.23 | 11.09 | 49.51 | 17.73 | 44.59 | 13.82 |
| VAL | 166.20 | 33.29 | 162.36 | 39.92 | 136.31 | 24.80 |
| ASA-Total | 0.20 | 0.09 | 0.19 | 0.10 | 0.17 | 0.16 |
| ADO | 0.27 | 0.15 | 0.3 | 0.18 | 0.31 | 0.16 |
| C0 | 25.02 | 7.06 | 21.98 | 6.11 | 24.26 | 4.39 |
| C10 | 0.08 | 0.06 | 0.07 | 0.16 | 0.06 | 0.03 |
| C10:1 | 0.04 | 0.03 | 0.04 | 0.04 | 0.03 | 0.01 |
| C10:2 | 0.00 | 0.01 | 0 | 0.00 | 0 | 0.00 |
| C2 | 8.20 | 2.07 | 8.16 | 2.28 | 7.96 | 2.00 |
| C3 | 1.57 | 0.58 | 1.41 | 0.37 | 1.08 | 0.52 |
| C3DC\C4OH | 0.05 | 0.02 | 0.04 | 0.01 | 0.04 | 0.01 |
| C4 | 0.10 | 0.07 | 0.12 | 0.06 | 0.11 | 0.04 |
| C4DC\C5OH | 0.38 | 0.13 | 0.38 | 0.13 | 0.35 | 0.10 |
| C5 | 0.08 | 0.03 | 0.08 | 0.03 | 0.07 | 0.02 |
| C5:1 | 0.01 | 0.00 | 0.01 | 0.00 | 0.01 | 0.00 |
| C5DC\C6OH | 0.04 | 0.01 | 0.04 | 0.01 | 0.04 | 0.01 |
| C6 | 0.02 | 0.01 | 0.02 | 0.02 | 0.02 | 0.01 |
| C6DC | 0.01 | 0.01 | 0.02 | 0.01 | 0.01 | 0.00 |
| C8 | 0.04 | 0.03 | 0.04 | 0.09 | 0.03 | 0.01 |
| C8:1 | 0.02 | 0.01 | 0.01 | 0.01 | 0.01 | 0.01 |
| D-ADO | 0.01 | 0.00 | 0.01 | 0.01 | 0 | 0.01 |
| C12 | 0.02 | 0.01 | 0.02 | 0.02 | 0.02 | 0.01 |
| C12:1 | 0.02 | 0.01 | 0.02 | 0.01 | 0.02 | 0.01 |
| C14 | 0.05 | 0.01 | 0.05 | 0.02 | 0.04 | 0.01 |
| C14:1 | 0.03 | 0.02 | 0.03 | 0.02 | 0.03 | 0.01 |
| C14:2 | 0.01 | 0.01 | 0.01 | 0.01 | 0.01 | 0.00 |
| C14OH | 0.00 | 0.00 | 0 | 0.00 | 0 | 0.00 |
| C16 | 0.84 | 0.19 | 0.75 | 0.22 | 0.75 | 0.23 |
| C16:1 | 0.05 | 0.01 | 0.04 | 0.01 | 0.04 | 0.01 |
| C16:1OH\C17 | 0.04 | 0.01 | 0.04 | 0.01 | 0.03 | 0.01 |
| C16OH | 0.01 | 0.00 | 0.01 | 0.00 | 0.01 | 0.00 |
| C18 | 0.47 | 0.15 | 0.46 | 0.14 | 0.42 | 0.14 |
| **C18:1** | **1.00** | **0.26** | **0.93** | **0.19** | **0.86** | **0.22** |
| C18:1OH | 0.01 | 0.00 | 0.01 | 0.00 | 0.01 | 0.00 |
| C18:2 | 0.23 | 0.07 | 0.21 | 0.08 | 0.17 | 0.06 |
| C18:2OH | 0.01 | 0.00 | 0.01 | 0.00 | 0.01 | 0.00 |
| C18OH | 0.00 | 0.00 | 0 | 0.00 | 0 | 0.00 |
| C20 | 0.02 | 0.01 | 0.02 | 0.01 | 0.02 | 0.01 |
| C20:0-LPC | 0.27 | 0.06 | 0.28 | 0.09 | 0.21 | 0.10 |
| C22 | 0.01 | 0.00 | 0.01 | 0.00 | 0.01 | 0.00 |
| C22:0-LPC | 0.18 | 0.05 | 0.18 | 0.06 | 0.14 | 0.05 |
| C24 | 0.02 | 0.01 | 0.02 | 0.01 | 0.02 | 0.01 |
| C24:0-LPC | 0.35 | 0.07 | 0.35 | 0.09 | 0.29 | 0.09 |
| C26 | 0.02 | 0.01 | 0.02 | 0.01 | 0.02 | 0.01 |

**Table S3.** Details of protein quantification in T1DM+ EVs *vs* HC EVs and T1DM- EVs *vs* HC EVs.

**Table S4.** Complete list of downstream and upstream predicted by IPA tool in the performed proteomics analyses.
